# An Account of a Remarkable Affection of the Testes

**Published:** 1797

**Authors:** Widdows Golding

**Affiliations:** Surgeon at Wallingford, in Berkshire, and Member of the Corporation of Surgeons in London.


V. *An Account of a remarkable Affection of the Testes. By Mr.* Widdows Golding, *Surgeon at Wallingford, in Berkshire, and Member of the Corporation of Surgeons in London. Communicated in a Letter to* William Babington, *M. D. Physician in London; and by him to Dr.* Simmons.

A SWELLING of the Testes has been mentioned by different writers* as a symptom of

* Vide Russell de Œconomia Naturæ in Morbis acutis et chronicis Glandularum. 8vo. 1755.—Rochard, Description of the *Cynanche parotidea*, or mumps, as the complaint is vulgarly called; and Sauvages, who has defcribed this difeafe in his *Nofologia Methodica* *, as a fpecies of catarrh, to which he has given the name of *Catarrhus Bellinfulanus*, from its having prevailed in Belle Ifle in 1757, points out this fwelling of the Teftes as a characteriftic mark by which this difeafe may be diftinguifhed from other fpecies of catarrh. But I have met with no writer who fpeaks of a fimilar affection of the Teftes, attended with pyrexia, and occurring without any previous fwelling of the parotid or maxillary glands. In the Summer and Autumn, however, of 1793, inftances of fuch fcription d'une Maladie particuliere des Glandes, endemique a Belle Ifle en mer. *Journal de Medecine.* 8vo. 1757.—Gooch, Cafes and Practical Remarks in Surgery. 8vo. 1758.—Cullen, Firft Lines of the Practice of Phyfic, Vol. I. 8vo. 1784.—Hamilton, Account of a Diftemper, by the common People in England vulgarly called the Mumps. *Tranfactions of the Royal Society of Edinburgh*, Vol. II. and *London Medical Journal*, Vol. XI.—M. Tiffot, in his work, entitled *Avis au Peuple*, (Tom. I. 8vo. Lyon, 1763, § 116) has alfo fpoken of the Mumps under the name of *Oreillons*; but he fays nothing of the affection of the Teftes.

* The defcription given of this complaint by M. Sauvages, is from the account communicated by M. Rochard to the Editor of the *Journal de Medecine*, and referred to in the preceding note.

an an affection, where the glands of the face or throat (except only in one of the cases) were not at all concerned, occurred to me, in this neighbourhood, in sufficient number, and with a correspondence of circumstances sufficiently striking to induce me to consider them as the effects of an epidemical state of the air.

As the form of the disease will, I think, best appear from a narrative of the several cases, I shall describe them to you in the order in which they occurred.

### CASE I.

I was desired, in the beginning of June, 1793, to visit a farmer's servant, about eighteen years of age, and of a plethoric habit, who had been ill four days. I found him in bed, labouring under the usual symptoms of fever, and complaining that his left testicle was much swelled, and gave him considerable pain; and that it had continued increasing in size and uneasiness since he was first taken; but what to impute it to he was at a loss to conceive, not having received the least external injury whatever. Upon examination I found it much enlarged and and inflamed, and the ſpermatic chord increaſed in ſize. I inquired if he had obſerved any diſcharge previouſly to its ſwelling, or had been troubled with any ſtrangury, not entertaining a doubt myſelf that the complaint was a Hernia humoralis, though the ſymptoms of fever were apparently more violent than they uſually are in ſuch caſes. I told him my ſuſpicions of the origin of the complaint; but he convinced me, by the account he gave of himſelf, and the examination he ſubmitted to, that they were without foundation. Excepting this appearance, he had not the ſlighteſt ſymptom that could be attributed to gonorrhœa or ſyphilis.

He was let blood; was directed to wear a bag truſs; to bathe the ſcrotum with *Aq. Lithargyr. acet. c.* and to take an emetic (compoſed of *Hydrargyr. Vitriol.* gr. v. and *Pulv. Ipecacuan.* gr. xv.) in the evening, and an active purgative the day following.

When I ſaw him again, a few days after, the ſymptoms of fever were abated; the teſticle was leſs painful, and the inflammation leſſened, although the part was not decreaſed in ſize; but there was ſome appearance of corrugation on the ſcrotum. The ſpermatic chord remained in the ſame ſtate as when I firſt examined it;

 but but this, with all the other appearances, gradually subsided, and within the space of about eighteen days from the commencement of the complaint, he was perfectly recovered.

### CASE II.

In the beginning of July, 1793, I was desired to visit A. L. a single man, about 23 years of age, by trade a butcher. I found him in bed, labouring under some slight symptoms of fever, though not accompanied with the same degree of inflammatory diathesis, as in Case I. He informed me (to use his own expression) that he had taken cold, and that it was settled in his left testicle. He had then been indisposed three days previously to my seeing him. Upon examining the part affected, I found the testicle much inflamed, enlarged, and very painful, putting on every appearance, externally, of an Hernia humoralis; but the spermatic chord was not so much enlarged as in Case I. I had reason to suspect, from the character of this man, that the complaint was syphilitic, although he declared the contrary. I examined him, therefore, with the strictest attention, but there was not the leaſt appearance of any chancre; he had no diſcharge or ſtrangury, nor any ſymptom whatever, that could lead me to conſider the caſe as venereal, excepting the ſtate of the teſticle. The mode of treatment adopted in Caſe I. was purſued in this, and the patient recovered in leſs time than the former.

### CASE III.

J. H. a ſingle man, about 20 years of age, whoſe employment was that of a gardener, applied to me about the ſame time as the laſt-mentioned patient. He complained of an enlargement of the left teſticle, which had been coming on for ſome days; and upon examination I found it increaſed in ſize, and conſiderably inflamed; it gave him great pain, and was attended with ſome degree of ſymptomatic fever. A careful examination, as in the former caſes, convinced me there was not the leaſt reaſon to ſuppoſe the complaint venereal; and he ſoon recovered, by purſuing the means adopted in the former caſes; but the affected teſticle has gradually become much ſmaller than the other.

### CASE IV.

J. P. a refpectable tradefman, of a corpulent habit, about 53 years of age, defired my attendance Aug. 15, 1793. He requefted me to examine his left tefticle, which he informed me had given him great pain for two days. I found it much inflamed, and confiderably fwelled; the chord was alfo enlarged, and he was affected with fome degree of fever.

Now whatever my fufpicions, when firft applied to, might be of the fingle men, whofe cafes I have defcribed, I could not for a moment entertain the fame ideas of this patient, who was a married man, of the ftricteft prudence and fobriety. There was no reafon here to fufpect any venereal infection; nor was there any external caufe to which the complaint could be afcribed. I adopted the fame mode of treatment as in the other cafes, excepting bleeding, as the patient defired this might, if poffible, be omitted, on account of a gouty affection to which he is fubject.

From the 15th to the 19th the pain, inflammation, and fwelling of the tefticle continued to increafe, accompanied with thirft, coftivenefs, nefs, cold chills, high-coloured urine, quick pulfe, lofs of appetite, and reftleffnefs.

On the 19th four leeches were applied to the fcrotum, and, after thefe, cloths dipped in a lotion compofed of Sal. Ammon. ℥i. Aq. Lythargyr. acet. c. ℔j. and Aq. Ammon. ℥j.

He was directed to take a mild purgative, and afterwards a faline draught, with half a drachm of tincture of antimony, every three hours; and a fimilar draught, with the addition of thirty drops of tincture of opium, at bed-time.

Aug. 20th. The inflammation and fwelling of the tefticle were but little abated, although the pain was not fo great. The opiate had procured reft, and the fymptomatic fever was leffened. He was defired to continue the ufe of the lotion and medicines.

22d. The tefticle this morning was lefs inflamed; the fkin of the fcrotum appeared corrugated; the pain was greatly abated; and the fymptomatic heat much decreafed. He was now directed to take the bark liberally.

28th. The tefticle was much leffened, and free from pain and inflammation.

30th. The tefticle appeared to be nearly of its natural fize, and the patient was fufficiently recovered to return to his bufinefs.

 CASE

### CASE V.

J. M. a reſpectable farmer in the neighbourhood of Wallingford, married, of a plethoric habit, and in the 55th year of his age, deſired my attendance Sept. 7, 1793. He had been confined a week, with a painful ſwelling of his right teſticle, but could give no cauſe for the complaint. Upon examination I found it enlarged, and attended with conſiderable inflammation; the ſpermatic chord was likewiſe increaſed in ſize, and the patient laboured under the uſual ſymptoms of beginning fever. The ſame medicines, and applications to the part, were had recourſe to as in the laſt-mentioned caſe.

Sept. 9th. The teſticle appeared much inflamed, and was more painful; the ſpermatic chord was more enlarged, and there was alſo an increaſe of fever. Four leeches were applied to the ſcrotum.

10th. The teſticle was leſs inflamed, and the ſkin ſomewhat corrugated, although the part was but little decreaſed in ſize. The ſymptoms of fever were abated.

12th. The inflammation, pain, and ſwelling of the teſticle were leſs, and the ſcrotum was more corrugated; but the ſpermatic chord was ſtill much enlarged. The feveriſh ſymptoms having ſubſided, he was directed to take the bark.

15th. He was ſo far recovered, as to be able to leave his room. The ſwelling and inflammation of the teſticle were much abated, but the chord ſtill remained enlarged.

17th. He was attacked yeſterday with a painful ſwelling of the right ſide of his face, previouſly to which he had cold rigors, ſucceeded by heat. The teſticle appeared much leſs, and free from inflammation.

23d. The ſwelling of the face had ſubſided; the teſticle and chord were nearly of their natural ſize, and the patient was in every reſpect pretty well recovered.

The late Mr. John Hunter, in his juſtly-celebrated Treatiſe on the Venereal Diſeaſe, (Part. III. chap. 13) relates inſtances of enlargement and inflammation of the teſticle, without any venereal infection; and I myſelf remember, four years ago, to have ſeen, in

St. Thomas's Hofpital, in London, a married man, fifty years old, who was admitted for the cure of a fuppofed Hernia humoralis, but who was perfectly free from every other appearance of venereal infection. The tefticle was confiderably enlarged and inflamed, and the complaint foon yielded to the mode of treatment commonly adopted in cafes of Hernia humoralis.

The inftances of this affection, which are more immediately the fubject of this paper, having all occurred to me between the months of June and September of the fame year, were probably of an epidemic nature, and as fuch I have thought they may be deemed worthy the attention of medical readers.

Dr. Wall, in a Differtation on the difeafes of the South-Sea Iflands, obferves, that "fwellings of the glands in the axilla and in-"guen, and even of the tefticle itfelf, are not "uncommon attendants of a great variety of "epidemic fevers, and difeafes communicated "by contagion, though not of a fyphilitic "nature*."

* *Vide* Differtations on Select Subjects in Chemiftry and Medicine; by Martin Wall, M. D. 8vo. Oxford, 1783. p. 158.

In

In the epidemic affection I have related, the diſeaſe was generally ten or twelve days before the part came to its full height of inflammation, and then the complaint gradually ſubſided, without putting on any appearance of ſuppuration or tendency to ſphacelus. In ſome of the patients the ſpermatic chord was larger than in others; and in none of them did it begin to decreaſe till the inflammation had abated, and the ſcrotum began to corrugate.

In one of the patients only, as I have already obſerved, has there been any perceptible waſting of the teſticle in conſequence of the complaint.

Three of the patients, as we have ſeen, were young, and ſingle men; the other two were married, and upwards of fifty years of age.

In one of the patients (Caſe V.) a ſwelling of the face came on when the affection of the teſticle was beginning to ſubſide; but I was inclined to think that this was an accidental circumſtance, ariſing from cold, and of courſe not connected with the original diſeaſe.

*Wallingford,*
*January* 17, 1795.

